# Diagnostic Value of ^11^C-Methionine (MET) and ^18^F-Fluorothymidine (FLT) Positron Emission Tomography in Recurrent High-Grade Gliomas; Differentiation from Treatment-Induced Tissue Necrosis

**DOI:** 10.3390/cancers4010244

**Published:** 2012-03-01

**Authors:** Hajime Shishido, Nobuyuki Kawai, Keisuke Miyake, Yuka Yamamoto, Yoshihiro Nishiyama, Takashi Tamiya

**Affiliations:** 1 Department of Neurological Surgery, Faculty of Medicine, Kagawa University, 1750-1 Miki-cho, Kita-Gun, Kagawa 761-0793, Japan; E-Mails: ranran@med.kagawa-u.ac.jp (H.S.); keisuke@med.kagawa-u.ac.jp (K.M.); tamiya@med.kagawa-u.ac.jp (T.T.); 2 Department of Radiology, Faculty of Medicine, Kagawa University, 1750-1 Miki-cho, Kita-Gun, Kagawa 761-0793, Japan; E-Mails: yuka@med.kagawa-u.ac.jp (Y.Y.); nisiyosi@med.kagawa-u.ac.jp (Y.N.)

**Keywords:** fluorothymidine (FLT), malignant glioma, methionine (MET), necrosis, positron emission tomography, recurrence

## Abstract

We retrospectively evaluated the usefulness of combined measurement of L-methyl-[^11^C]methionine (MET) and 3'-deoxy-3'-[^18^F]fluorothymidine (FLT) positron emission tomography (PET) in the differential diagnosis between recurrent gliomas and necrotic lesions. Twenty-one patients with high-grade glioma, previously treated with surgery and radiotherapy with chemotherapy and first radiological suspicion of recurrence were enrolled. The uptake was assessed by the maximum standardized uptake value (SUVmax) and lesion-to-normal tissue count density ratio (L/N ratio). Of the 21 lesions, 15 were diagnosed recurrent gliomas and six were necrotic lesions. The average SUVmax was not significantly different between recurrent gliomas and necrotic lesions on either MET-PET or FLT-PET. The average L/N ratio of recurrent gliomas (3.36 ± 1.06) was significantly higher than that of necrotic lesions (2.18 ± 0.66) on MET-PET (*p* < 0.01) and the average L/N ratio of recurrent gliomas (7.01 ± 2.26) was also significantly higher than that of necrotic lesions (4.60 ± 1.23) on FLT-PET (*p* < 0.01). ROC curve analysis showed that the areas under the curves were high but not different between MET- and FLT-PET. PET studies using MET and FLT are useful in the differentiation of recurrent glioma from treatment-induced necrotic lesion. However, there is no complementary information in the differentiation with simultaneous measurements of MET- and FLT-PET.

## 1. Introduction

Recurrence in glioma may occur after macroscopic total removal of tumor or stabilization of tumor with treatment. In many cases, recurrence occurs at the initial tumor bed or within 2 cm of the primary site [1]. Radiation injury and postoperative changes are also usually found around the primary site. Moreover, radiation necrosis and recurrent tumor frequently coexist [2]. Differentiation between recurrent tumor and necrotic tissue changes is crucial because the two entities require different treatment strategies and have a completely different prognosis. Treatment-induced necrosis can produce disruption in the blood-brain barrier (BBB) by endothelial damage, and thus contrast enhancement and edema are indistinguishable from recurrent tumor on conventional magnetic resonance imaging (MRI) [3].

Positron emission tomography (PET) can overcome the limitation of conventional MRI and can be used to derive information on tumor metabolism. PET with L-methyl-[^11^C]methionine (MET) is a well-established imaging tool for brain tumour detection [4], tumour grading [5,6,7], prediction of prognosis [6,8,9], evaluation of response to treatment [10,11,12,13], and differentiation between tumor recurrence and radiation necrosis [14,15] in patients with gliomas. However, increased MET uptake in non-neoplastic lesions including inflammation, infarction, or hemorrhage may cause a false positive result [16,17]. The short half-life of ^11^C (20 min) and rapid *in vivo* degradation also make MET-PET less useful for routine clinical use. A fluorinated thymidine analog, 3'-deoxy-3'-[^18^F]fluorothymidine (FLT), has emerged as a promising PET tracer for evaluating tumor proliferating activity in various malignant brain tumors [18,19,20]. FLT is phosphorylated by thymidine kinase-1 (TK1), a principle enzyme in the salvage pathway of DNA synthesis, and trapped inside the cells. Phosphorylated FLT appears resistant to degradation and is suitable for imaging with PET. The application of FLT phosphorylation as a marker of cell proliferation is based on the assumption that cellular FLT trapping is a representation of thymidine incorporation into DNA [21,22]. As FLT uptake in the normal brain tissue is very low, FLT-PET provides a low-background brain image and thus is considered to be an ideal PET tracer for the imaging of brain tumors. FLT-PET has been found useful for noninvasive grading of newly diagnosed gliomas [7,18,19,20,21,23]. However, there is some data on the value of FLT-PET in the evaluation of recurrent brain tumors [24,25]. A recent study shows that FLT-PET has a high sensitivity but a low specificity, which has a limited role in the diagnosis of recurrent gliomas [25]. This retrospective study was conducted to evaluate the usefulness of combined use of MET-PET and FLT-PET in the differential diagnosis between recurrent gliomas and treatment-induced necrotic lesions.

## 2. Results and Discussion

### 2.1. Results

#### 2.1.1. Visual Assessment

Of 21 lesions, 15 were recurrent tumors (initial diagnosis; eight glioblastomas, three anaplastic oligoastrocytomas, two anaplastic astrocytomas, one gliosarcoma, and one gliomatosis cerebri) and six were necrotic lesions by the final diagnosis. All lesions showed moderate to strong enhancement effect on T1-weighted MR image after gadolinium administration.

**Figure 1:**
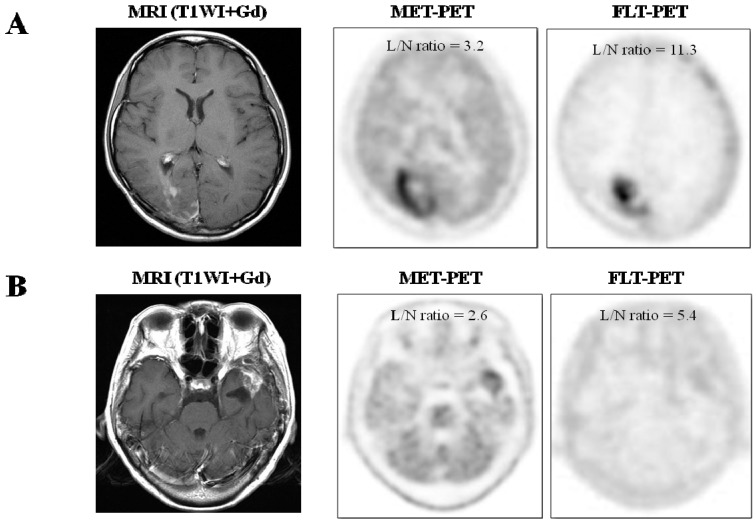
(**A**) Imaging of a 22 year-old female (case 9) with glioblastoma, previously treated with tumor resection followed by conventional radiotherapy and temozolomide. T1-weighted contrast enhanced MR image showing irregular enhancement in the right occipital lobe. MET-PET and FLT-PET show intense uptake of tracer in the lesion. Recurrent glioblastoma was pathologically confirmed by surgery; (**B**) Imaging of 61 year-old female (case 21) with glioblastoma, previously treated with tumor resection followed by conventional radiotherapy and temozolomide. T1-weighted contrast enhanced MR image showing enhanced mass in the left temporal lobe. MET-PET shows mild uptake of tracer and FLT-PET shows faint uptake of tracer in the lesion. Necrosis and gliosis dominant tissue was pathologically confirmed by surgery.

All 15 recurrent gliomas showed increased uptake on FLT- and MET-PET (Figure 1A). Of the six necrotic lesions, all showed increased uptake on FLT-PET and five lesions showed increased uptake on MET-PET (Figure 1B). The MET uptake of one necrotic lesion (Case 19) was faint compared to the normal brain parenchyma (L/N ratio of 1.25).

#### 2.1.2. Semi-Quantitative Analysis

**Figure 2:**
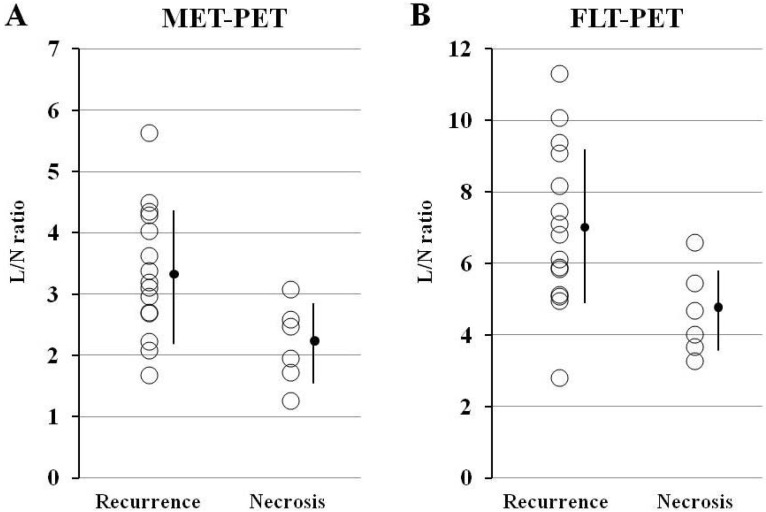
Comparisons of L/N ratios for MET (**A**) and FLT (**B**) in cases of recurrent gliomas (Recurrence) and necrotic lesions (Necrosis). (**A**) Although there is a significant overlap of tracer uptake, the average MET L/N ratio in recurrent gliomas is significantly higher (*p* < 0.01) compared with that in necrotic lesions. (**B**) Again, there is a significant overlap of tracer uptake, and the average FLT L/N ratio in recurrent gliomas is significantly higher (*p* < 0.01) compared with that in necrotic lesions.

The average SUVmax of 15 recurrent gliomas (4.59 ± 1.64) was higher than that of six necrotic lesions (3.51 ± 0.83) on MET-PET but this was not statistically significant (*p* = 0.063). The average L/N ratio of recurrent gliomas (3.36 ± 1.06) was significantly higher than that of necrotic lesions (2.18 ± 0.66) on MET-PET (*p* < 0.01) (Figure 2A). The average SUVmax of 15 recurrent gliomas (1.46 ± 0.64) was higher than that of six necrotic lesions (1.01 ± 0.45) on FLT-PET but this was not statistically significant (*p* = 0.095). The average L/N ratio of recurrent gliomas (7.01 ± 2.26) was significantly higher than that of necrotic lesions (4.60 ± 1.23) on FLT-PET (*p* < 0.01) (Figure 2B).

**Figure 3:**
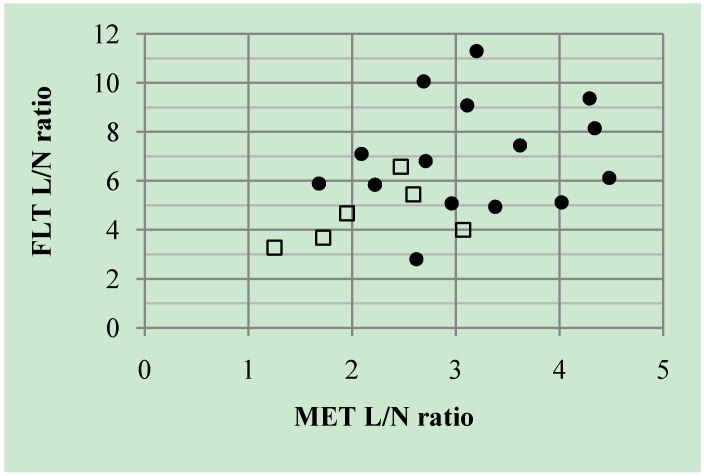
Linear regression analysis of L/N ratio between MET and FLT. There is no significant correlation between the individual L/N ratio of MET and FLT (*r* = 0.41, *p* = 0.064). Closed circles (●) indicate the cases with recurrent glioma and open squares (□) indicate the cases with necrotic lesion.

All patients underwent MET- and FLT-PET. The mean duration between the two PET examinations was 3.9 ± 4.9 days (range, 1–20 days). Significant correlation was not observed between the individual L/N ratio of MET and FLT (*r* = 0.41, *p* = 0.064) (Figure 3). There was no complementary information in the differential diagnosis with simultaneous measurement of MET- and FLT-PET.

**Figure 4:**
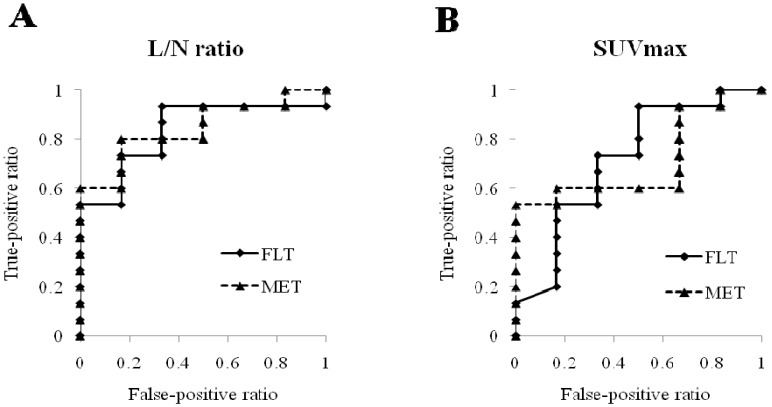
ROC curve indicating that the areas under the curves are not different between tumor uptake values of MET and FLT in both L/N ratio (**A**) and SUVmax (**B**).

Analysis of the ROC curve showed that the areas under the curves were high but not different between tumor uptake values of MET and FLT in both L/N ratio (Az: 0.844 for MET and 0.833 for FLT) (Figure 4A) and SUVmax (Az: 0.711 for MET and 0.717 for FLT) (Figure 4B). When we set the L/N ratio threshold on MET-PET at 2.69, the sensitivity of MET-PET for differential diagnosis was 80% and the specificity was 83%. Again, when we set the L/N ratio threshold on FLT-PET at 4.94, the sensitivity of FLT-PET for differential diagnosis was 93% and the specificity was 67%.

### 2.2. Discussion

Contrast enhancement of brain tumors depends on the BBB breakdown. Treatment-induced necrosis or necrosis occurring spontaneously during tumor progression may also show contrast enhancement and hence cannot be distinguished accurately from tumor recurrence after treatment [3]. In addition, contrast enhancement may be substantially reduced by steroid treatment for reducing brain edema [26]. Morphological imaging often does not adequately reflect the underlying tumor biology and its metabolic activity imposes considerable demand for the development of alternative imaging modalities to adequately characterize the lesions.

Labeled amino acids and their analogs are particularly attractive for imaging brain tumors because of the high uptake in tumor tissue and low uptake in normal brain parenchyma and, as a consequence, higher tumor-to-normal tissue contrast. The most frequently used radiolabeled amino acid, ^11^C-MET, can be applied only in centers with an on-site cyclotron because of short half-life of ^11^C (20 min) and rapid *in vivo* degradation. MET-PET possesses high specificity in tumor detection [4], tumor delineation [27], and differentiation of benign from malignant lesions [4,28]. Various studies have found a correlation between tumor grade and MET uptake in gliomas [5,6,7]. MET readily transports the intact BBB through neutral amino acid transporters and is incorporated into the area with active tumor. MET uptake in gliomas significantly correlates with cell proliferation, *in vitro* Ki-67 expression [7,29,30]. Increased MET uptake seems to be caused by increased carrier-mediated and passive transport rather than elevated protein synthesis and is highly correlated with microvessel density (angiogenesis) in gliomas [30,31]. In an earlier study on suspected glioma recurrence, there was no significant relationship between primary tumor histopathology (grade) and MET uptake, in contrast to the findings of this study performed in a pre-therapeutic setting [9]. Also, no significant difference was found in MET uptake between glioma recurrence and radiation necrosis following the stereotactic radiosurgery [32]. Although no significant difference was found in MET SUVmax between recurrent gliomas and treatment-induced necrotic lesions in our study, the average L/N ratio of recurrent gliomas was significantly higher than that of the treatment-induced lesions on MET-PET (*p* < 0.01). MET SUVmax measurement might be uninformative because of large interindividual variability of large neutral amino acids in the plasma competing with MET at the amino acid transporter. In this study, there was a significant overlap of MET uptake between glioma recurrence and radiation necrosis. It has been reported that gliomas with oligodendroglial components show relatively high MET uptake even in low grade gliomas [7]. Moreover, radiation necrosis and recurrent tumor frequently coexist [2]. Recently, Terakawa *et al*. [15] showed that quantitative analysis of MET-PET data is useful in the differentiation of glioma recurrence from radiation necrosis, and an L/N ratio of 1.58 provides the best sensitivity (75%) and specificity (75%). Our study showed higher sensitivity (80%) and lower specificity (83%) for differential diagnosis, but the quantitative values did not appear to be absolute indicators.

^18^F-FLT is a PET tracer that allows for noninvasive assessment of tumor proliferation [18,19,20]. In contrast to MET, which provides only an indirect measure of proliferation status as amino acid uptake in the tumor, FLT allows the direct measurement of cellular TK1 activity, which has been reported to be proportional to the proliferation activity of the tumor [33]. In our previous study, a linear regression analysis indicated a much more significant correlation of the Ki-67 index with FLT uptake than with MET uptake in gliomas [7]. As uptake of FLT is low in intact brain tissue, FLT-PET provides a low-background cerebral image and thus is considered to be an ideal and attractive PET tracer for the imaging of gliomas [18]. At the beginning of this study, we expected that FLT-PET to be specific for the differential diagnosis of treatment-induced tissue necrosis from tumor recurrence and possibly helpful in cases with a false-positive result on MET-PET, because FLT uptake reflects the proliferation activity of viable lesions. However, the specificity of FLT-PET in our study was not statistically different from the specificity of MET-PET. FLT cannot be transported well across the intact BBB, but it can pass through the disrupted BBB of not only tumorous lesions but also benign lesions such as inflammation and tissue necrosis [34]. The non-specific uptake of FLT in treatment-induced necrosis may largely depend on the BBB breakdown. Therefore, simple measures of FLT uptake, such as SUVmax, can lead to incorrect interpretation. A recent study shows that FLT-PET has a high sensitivity but a lower specificity in accordance with our results, which has a limited role in the diagnosis of recurrent gliomas [25]. To increase the sensitivity of FLT-PET, separating transport effect from tissue retention of metabolically trapped FLT could be helpful. In human FLT-PET studies, kinetic modeling enables distinction between increased FLT uptake due to increased transport through the BBB and increased uptake due to an increased phosphorylation by TK1 [23,35]. A previous study in newly diagnosed and recurrent gliomas of different grades showed that the major proportion of FLT uptake into the tumor is due to increased transport and influx [23]. Kinetic analysis requires sequential arterial blood sampling and dynamic PET scanning requires a long acquisition time (60 min at our institution), so it is difficult to perform on all the cases as routine clinical practice. Schiepers *et al*. [36] proposed a less invasive kinetic analysis without arterial blood sampling. This may increase the clinical use of FLT-PET kinetic analysis in the differential diagnosis between recurrent tumors and treatment-induced necrotic lesions.

Modern MRI techniques are being increasingly used to improve the sensitivity and specificity of tumor diagnostics; these techniques offer possibilities for the detection of functional information. MR spectroscopy (MRS) can assess tumor metabolism and physiology and perfusion-weighted imaging (PWI) can assess vascular integrity and function. Histopathologically, radiation necrosis is characterized by endothelial damage and fibrinoid necrosis, whereas recurrent tumor is characterized by increased microvasculature. A recent study showed that regional cerebral blood volume (rCBV) measurements to be equivalent to MET-PET in the follow-up of patients with high-grade gliomas [37]. No studies comparing PWI and FLT-PET in the differential diagnosis between recurrent tumors and treatment-induced necrotic lesion has been published in the literatures.

This study has several limitations. It has a small number of treatment-induced necrotic lesions (n = 6) and the final diagnosis was made by clinical and radiological follow-up in eight of 21 cases. When we evaluate 13 patients (10 recurrent gliomas and three necrotic lesions) whose diagnosis was confirmed with histopathology, the results are similar to those in all patients. The average SUVmax was not significantly different between recurrent gliomas and necrotic lesions on either MET-PET or FLT-PET. The average T/N ratio of recurrent gliomas (3.70 ± 0.96) was significantly higher than that of necrotic lesions (2.54 ± 0.56) on MET-PET (*p* < 0.05) and the average T/N ratio of recurrent gliomas (7.03 ± 2.51) was significantly higher than that of necrotic lesions (4.70 ± 0.72) on FLT-PET (*p* < 0.05). Again, the number is small and the results are preliminary. Further studies involving a larger number of treatment-induced lesions and kinetic analysis are necessary for accurate evaluation of the specificity of FLT-PET.

## 3. Experimental Section

### 3.1. Patients

Twenty-one consecutive patients (11 men, 10 women; mean age: 54.0 ± 13.6 years; range: 22–71 years) were included in this retrospective study (Table 1). Inclusion criteria were histopathologically proven high grade (III and IV) glioma, previously treated with surgery (subtotal or partial removal), radiotherapy and temozolomide (concomitant and adjuvant) and first radiological suspicion of recurrence during their follow-up. Histopathological diagnosis was obtained after PET examinations in 13 patients. Diagnosis was made by clinical and radiological follow-up in the other eight patients at the time of this study. When the patient showed progressive enlargement of the lesion with increased brain edema, we considered the lesion was tumor recurrence. All the patients except who were diagnosed as treatment-induced necrotic lesion by surgery continued temozolomide treatment. Patients who were diagnosed as tumor recurrence by observation died within 2–8 months after the examination. On the other hand, patients who were diagnosed as necrotic lesion survived 10–77 months after the examination.

**Table 1:** Summary of demographic and imaging data of the patients.

Case	Age	Sex	Primary tumor histopathology	MET		FLT		Method * (months)	Diagnosis
				SUVmax	L/N ratio	SUVmax	L/N ratio		
1	49	F	Anaplastic astrocytoma	2.80	2.22	1.11	5.84	Surgery	Recurrence
2	58	M	Anaplastic astrocytoma	3.44	2.96	0.66	5.08	Surgery	Recurrence
3	71	M	Anaplastic oligoastrocytoma	5.17	4.34	1.06	8.15	Surgery	Recurrence
4	66	F	Anaplastic oligoastrocytoma	5.58	3.38	0.84	4.94	Observation (8) D	Recurrence
5	44	F	Anaplastic oligoastrocytoma	5.59	4.02	0.87	5.12	Surgery	Recurrence
6	59	M	Gliomatosis cerebri	2.69	2.09	1.49	7.10	Observation (4) D	Recurrence
7	59	M	Glioblastoma	2.93	2.69	1.71	10.06	Observation (2) D	Recurrence
8	71	F	Glioblastoma	2.58	1.68	1.65	5.89	Observation (2) D	Recurrence
9	22	F	Glioblastoma	5.21	3.20	3.05	11.30	Surgery	Recurrence
10	30	F	Glioblastoma	5.50	3.11	2.27	9.08	Surgery	Recurrence
11	35	M	Glioblastoma	8.66	5.62	0.84	2.8	Surgery	Recurrence
12	51	M	Glioblastoma	5.56	4.48	1.04	6.12	Surgery	Recurrence
13	60	M	Glioblastoma	5.28	4.29	1.78	9.37	Surgery	Recurrence
14	55	F	Glioblastoma	3.41	2.71	1.84	6.81	Observation (5) D	Recurrence
15	54	M	Gliosarcoma	4.45	3.62	1.64	7.45	Surgery	Recurrence
16	52	F	Anaplastic astrocytoma	2.61	1.72	0.55	3.67	Observation (70) S	Necrosis
17	39	M	Anaplastic astrocytoma	3.54	2.47	1.84	6.57	Observation (33) S	Necrosis
18	69	F	Glioblastoma	3.61	1.95	1.12	4.67	Surgery	Necrosis
19	68	M	Glioblastoma	2.52	1.25	0.82	3.28	Observation (10) S	Necrosis
20	62	M	Glioblastoma	4.64	3.07	0.76	4.00	Surgery	Necrosis
21	61	F	Glioblastoma	4.14	2.59	0.98	5.44	Surgery	Necrosis

* S: survived; D: dead.

### 3.2. ^11^C-MET and ^18^F-FLT Synthesis and PET Acquisition

MET and FLT were produced using HM-18 cyclotron (Sumitomo Heavy Industries Co., Tokyo, Japan). FLT was synthesized using the method described by Machulla *et al*. [38] and the radiochemical purity of the produced FLT was >95%. MET was produced by proton bombardment of ^14^N_2_. The resultant ^11^CO_2_ was reduced to ^11^C-methanol by lithium aluminum hydride and subsequently converted to ^11^C-CH_3_I by the addition of hydrogen iodide by a modified method of Ishiwata *et al*. [39] and the radiochemical purity of the produced MET was >99%. PET examinations were performed using an ECAT EXACT HR+ scanner (Siemens/CTI, Knoxville, TN, USA) in the three-dimensional acquisition mode. The image system enabled the simultaneous acquisition of 51 transverse per field of views (FOVs), with intersection spacing of 3 mm, for a total axial FOV of 15 cm. The in-plane reconstructed resolution was 4.7 mm full-width at half maximum in the brain FOV.

No special dietary instructions were given to the patients before PET examination. Images were acquired with patients in the supine position, resting, with their eyes closed. Using ^68^Ge rod sources rotating around the head, transmission images of the brain were obtained for 3 min for MET-PET and for 5 min for FLT-PET. A dose of 126–318 MBq (mean, 215 ± 58 MBq) of ^11^C-MET or 91–337 MBq (mean, 204 ± 79 MBq) of ^18^F-FLT was injected intravenously. Regional emission images of the brain were obtained for 5 min, beginning 10 min after the ^11^C-MET injection and for 10 min, beginning 40 min after the ^18^F-FLT injection.

### 3.3. Data Analysis

MET and FLT uptakes in the brain lesion were semi-quantitatively assessed by evaluating the standardized uptake value (SUV). The region of interest (ROI) was set manually by an observer around the hottest area of each lesion. The maximum value of SUV (SUVmax) was regarded as the representative value of each lesion. To calculate the lesion-to-normal tissue count density (L/N) ratios, ROI was set on the normal brain parenchyma (usually contralateral normal cerebral tissue excluding ventricles) and the mean value of SUV (SUVmean) was calculated. The L/N ratio was determined by dividing the SUVmax of the lesion with the SUVmean of the normal brain tissue.

### 3.4. Statistical Analysis

All parametric data were expressed as mean ± SD. The Wilcoxon signed rank test was used to evaluate the difference between MET and FLT in brain lesions. The Mann-Whitney U test was used to evaluate the difference between SUVmax and the L/N ratio of recurrent gliomas and of necrotic lesions. Linear regression analysis was performed to evaluate the relationship between MET and FLT uptake in the lesion. Receiver-operating-characteristics (ROC) curve analysis was used to determine the best index of MET-PET and FLT-PET and compare the differentiation capability between tumor uptake of MET and FLT. *P* < 0.05 was considered statistically significant.

## 4. Conclusion

PET studies using MET and FLT are useful in the differentiation of recurrent high-grade gliomas from treatment-induced necrotic lesions. However, quantitative values do not appear to be absolute indicators and no complimentary information is provided in the differentiation with simultaneous measurements of MET-PET and FLT-PET.
